# Web GIS in practice X: a Microsoft Kinect natural user interface for Google Earth navigation

**DOI:** 10.1186/1476-072X-10-45

**Published:** 2011-07-26

**Authors:** Maged N Kamel Boulos, Bryan J Blanchard, Cory Walker, Julio Montero, Aalap Tripathy, Ricardo Gutierrez-Osuna

**Affiliations:** 1 Faculty of Health, University of Plymouth, Drake Circus, Plymouth, Devon PL4 8AA, UK; 2 Department of Computer Science and Engineering, Texas A&M University, College Station, TX 77843-3112, USA

## Abstract

This paper covers the use of depth sensors such as Microsoft Kinect and ASUS Xtion to provide a natural user interface (NUI) for controlling 3-D (three-dimensional) virtual globes such as Google Earth (including its Street View mode), Bing Maps 3D, and NASA World Wind. The paper introduces the Microsoft Kinect device, briefly describing how it works (the underlying technology by PrimeSense), as well as its market uptake and application potential beyond its original intended purpose as a home entertainment and video game controller. The different software drivers available for connecting the Kinect device to a PC (Personal Computer) are also covered, and their comparative pros and cons briefly discussed. We survey a number of approaches and application examples for controlling 3-D virtual globes using the Kinect sensor, then describe Kinoogle, a Kinect interface for natural interaction with Google Earth, developed by students at Texas A&M University. Readers interested in trying out the application on their own hardware can download a Zip archive (included with the manuscript as additional files 1, 2, &3) that contains a 'Kinnogle installation package for Windows PCs'. Finally, we discuss some usability aspects of Kinoogle and similar NUIs for controlling 3-D virtual globes (including possible future improvements), and propose a number of unique, practical 'use scenarios' where such NUIs could prove useful in navigating a 3-D virtual globe, compared to conventional mouse/3-D mouse and keyboard-based interfaces.

## Background

### What is Kinect?

Launched in November 2010, Kinect is a motion sensing USB (Universal Serial Bus) input device by Microsoft that enables users to control and naturally interact with games and other programs without the need to physically touch a game controller or object of any kind. Kinect achieves this through a natural user interface by tracking the user's body movement and by using gestures and spoken commands [1,2]. Kinect holds the Guinness World Record as the fastest selling consumer electronics device, with sales surpassing 10 million units as of 9 March 2011 [3].

Kinect uses technology by Israeli company PrimeSense that generates real-time depth, colour and audio data of the living room scene. Kinect works in all room lighting conditions, whether in complete darkness or in a fully lit room, and does not require the user to wear or hold anything [4,5] (*cf*. Sony's PlayStation Move and Nitendo Wii Remote controllers). PrimeSense also teamed up with ASUS to develop a PC-compatible device similar to Kinect, which they called ASUS Xtion and launched in the second quarter of 2011 [2,6].

**Figure 1:**
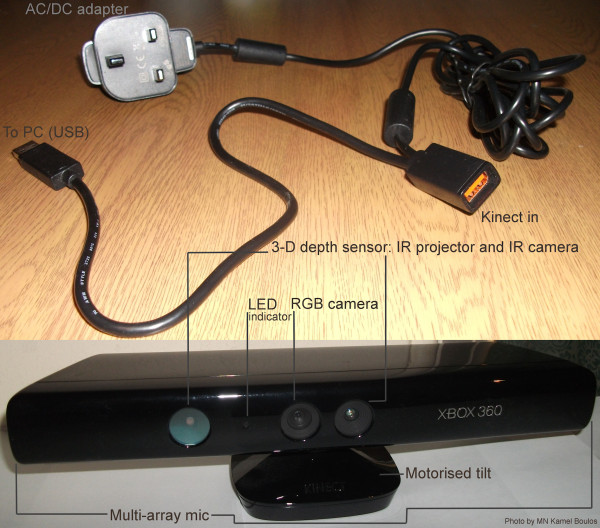
**Anatomy of Microsoft Kinect**.

Kinect is a horizontal bar connected to a small base with a motorised pivot (to follow the user around, as needed), and is designed to be positioned lengthwise above or below the computer or TV screen (Figure 1). The device features an RGB (Red Green Blue) colour camera, a depth sensor (using an infrared--IR projector and an IR camera), and a noise-cancelling, multi-array microphone (made of four microphones, which can also contribute to detecting a person's location in 3-D (three-dimensional) space) [5,7]. Kinect also incorporates an accelerometer (probably used for inclination and tilt sensing, and possibly image stabilisation [7]). Running proprietary firmware (internal device software), these components together can provide full-body 3-D motion capture, gesture recognition, facial recognition, and voice recognition capabilities [2,4]. (Functions, accuracy and usability will also greatly depend on device drivers and associated software running on the host machine (which can be a Windows, Mac or Linux PC, or an Xbox 360 game console) and used to access the Kinect hardware--see discussion about drivers below.)

Kinect is capable of simultaneously tracking two active users [2,8]. For full-body, head to feet tracking, the recommended user distance from the sensor is approximately 1.8 m for a single user; when there are two people to track at the same time, they should stand approximately 2.5 m away from the device. Kinect requires a minimum user height of 1 m (standing distance and user height figures are according to information printed on the Microsoft Kinect retail box).

Because Kinect's motorised tilt mechanism requires more power than what can be supplied via USB ports, the device makes use of a proprietary connector and ships with a special power supply cable that splits the connection into separate USB and power connections, with power being supplied from the mains by way of an AC/DC adapter (Figure 1).

Kinect can be bought new in the UK for less than £100 per unit (http://Amazon.co.uk consumer price in GBP, including VAT, as of July 2011).

### PC driver support

In December 2010, OpenNI and PrimeSense released their own Kinect open source drivers and motion tracking middleware (called NITE) for PCs running Windows (7, Vista and XP), Ubuntu and MacOSX [9,10]. FAAST (Flexible Action and Articulated Skeleton Toolkit) is a middleware developed at the University of Southern California (USC) Institute for Creative Technologies that aims at facilitating the integration of full-body control with virtual reality applications and video games when using OpenNI-compliant depth sensors and drivers [11,12].

**Figure 2:**
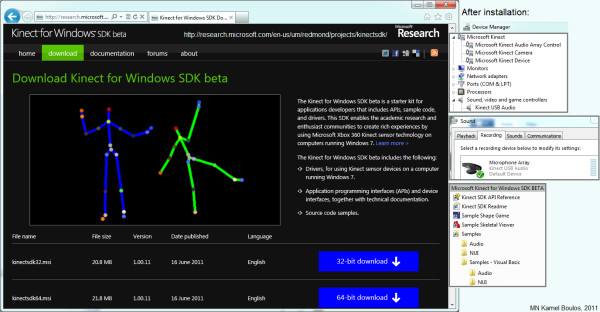
**Microsoft Kinect for Windows SDK beta (non-commercial, June 2011 release)**.

In June 2011, Microsoft released a non-commercial Kinect Software Development Kit (SDK) for Windows that includes Windows 7-compatible PC drivers for the Kinect device (Microsoft's SDK does not support older Windows versions or other operating systems) [13]. Microsoft's SDK allows developers to build Kinect-enabled applications in Microsoft Visual Studio 2010 using C++, C# or Visual Basic (Figure 2). Microsoft is planning to release a commercial version of the Kinect for Windows SDK with support for more advanced device functionalities [2].

There is also a third set of Kinect drivers for Windows, Mac and Linux PCs by the OpenKinect (libFreeNect) open source project [14]. Code Laboratories' CL NUI Platform offers a signed driver and SDK for multiple Kinect devices on Windows XP, Vista and 7 [15].

The three sets of drivers vary greatly in their affordances. For example, the official Microsoft Kinect for Windows SDK does not need a calibration pose; the 'Shape Game' demo supplied with the SDK works impressively well (for one or two active users) immediately after installing the SDK, without the need to calibrate user pose [8]. This contributes to an excellent Kinect digital Out-Of-Box Experience (OOBE) for PC users that is comparable to the OOBE offered to Kinect Xbox 360 game console users [16]. However, Microsoft Kinect for Windows SDK beta (June 2011 release) does not offer finger/hand gesture recognition or hands-only tracking and is limited to skeletal tracking. OpenNI drivers are more flexible in this respect, but on the negative side, they require a calibration pose and lack the advanced audio processing (speech recognition) that is provided by Microsoft's official SDK [17-20].

### Applications beyond playing games

Many developers and research groups around the world are exploring possible applications of Kinect (and similar devices such as ASUS Xtion [6]) that go beyond the original intended purpose of these sensors as home entertainment and video game controllers [2,21,22]. These novel applications include 3-D and enhanced video teleconferencing (e.g., the work done by Oliver Kreylos at the University of California Davis combining two Kinect devices [23,24], and the 'Kinected Conference' research by Lining Yao, Anthony DeVincenzi and colleagues at MIT Media Lab to augment video imaging with calibrated depth and audio [25,26]); using Kinect to assist clinicians in diagnosing a range of mental disorders in children (research by Nikolaos Papanikolopoulos and colleagues at the University of Minnesota [27]); and a more practical use of the device to control medical imaging displays during surgery without having to physically touch anything, thus reducing the chance of hand contamination in operating theatres (e.g., the work conducted within the Virtopsy Project at the Institute of Forensic Medicine, University of Bern, Switzerland [28], and the system currently in use by Calvin Law and his team at Toronto's Sunnybrook Health Sciences Centre [29], as well as the demonstration of a similar concept by InfoStrat, a US-based IT services company [30]).

PC-based Kinect applications have also been developed for controlling non-gaming 3-D virtual environments (3-D models, virtual worlds and virtual globes), e.g., [31,32]. These environments share many common features with 3-D video games, the original target domain of the Kinect sensor. Evoluce, a German company specialising in natural user interfaces, offers a commercial solution for multi-gesture interaction using Kinect's 3-D depth sensing technology under Windows 7 [33]. Thai Phan at the University of Southern California Institute for Creative Technologies wrote software based on the OpenNI toolkit to control a user's avatar in the 3-D virtual world of Second Life^® ^and transfer the user's social gestures to the avatar in a natural way [34].

**Figure 3:**
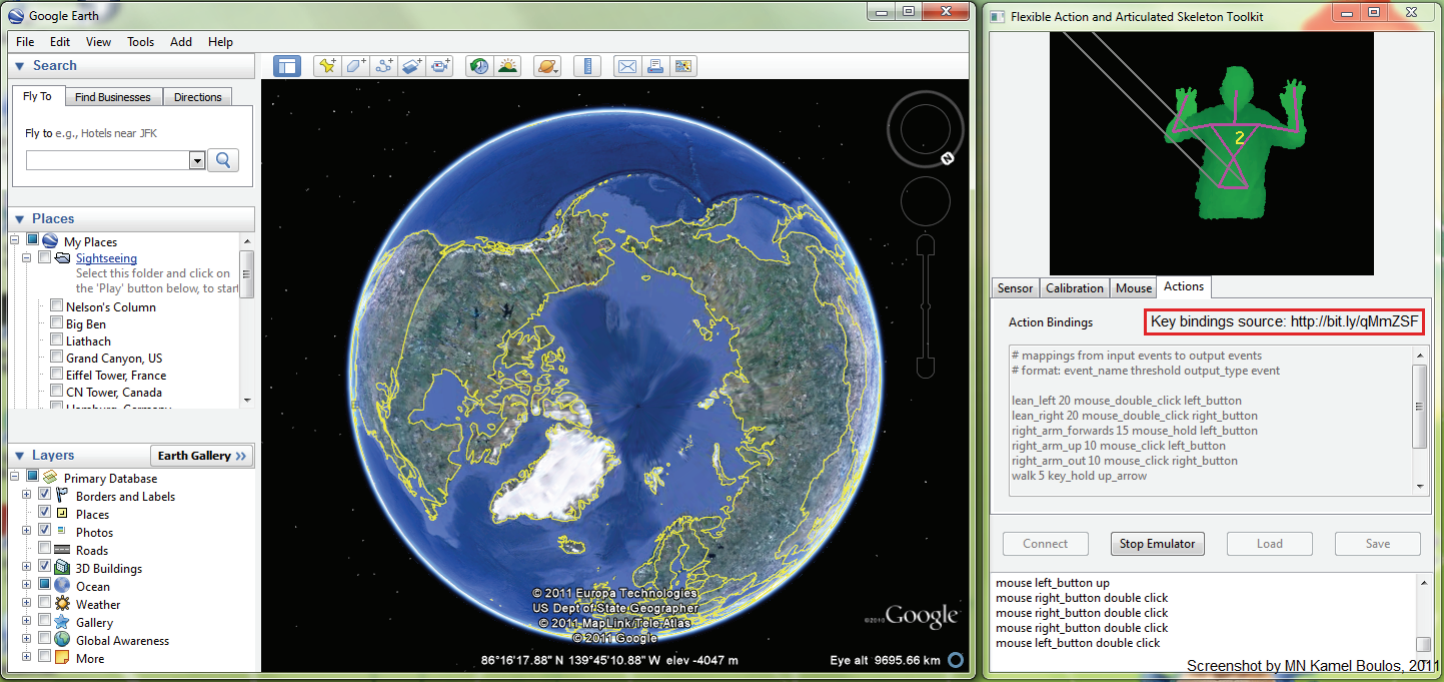
**Using FAAST with suitable 'key bindings' to navigate Google Earth and Street View**.

FAAST [11] has been used with suitable 'key bindings' (to map user's movement and gestures to appropriate keyboard and mouse actions) to navigate Google Earth and Street View (Figure 3) [35,36]. InfoStrat recently demonstrated the use of their Motion Framework [37] to control Bing Maps with a Kinect sensor [38]. They also used custom Bing Maps-powered GIS (Geographic Information System) and data visualisation applications to showcase their Motion Framework's ability to work with multi-modal input from motion sensing (such as Microsoft Kinect), multi-touch, speech recognition, stylus, and mouse devices [39,40]. Building on InfoStrat's work (which brought multi-touch gestures such as pinch and zoom to Kinect), Response Ltd, a small Hungarian company, developed and demonstrated an alternative solution to navigate Bing Maps using the Kinect sensor, which, they claim, offers a more 'Kinect-like experience' by allowing the user to use his/her whole body to control the map [41,42].

## Kinoogle: a Kinect interface for natural interaction with Google Earth

In this paper, we introduce Kinoogle, a natural interaction interface for Google Earth using Microsoft Kinect. Kinoogle allows the user to control Google Earth through a series of hand and full-body gestures [43,44]. We begin by describing the software design and modules underlying Kinoogle, then offer detailed user instructions for readers interested in trying it out.

### Hardware and third-party software

The main hardware used in Kinoogle is Microsoft Kinect. Kinect generates a depth map in real time, where each pixel corresponds to an estimate of the distance between the Kinect sensor and the closest object in the scene at that pixel's location. Based on this map, the Kinect system software allows applications such as Kinoogle to accurately track different parts of a human body in three dimensions. Kinoogle has been designed around a specific setup for operation that includes the user standing about one metre in front of Kinect, with shoulders parallel to the device, and the sensor placed at the height of the user's elbows.

**Figure 4:**
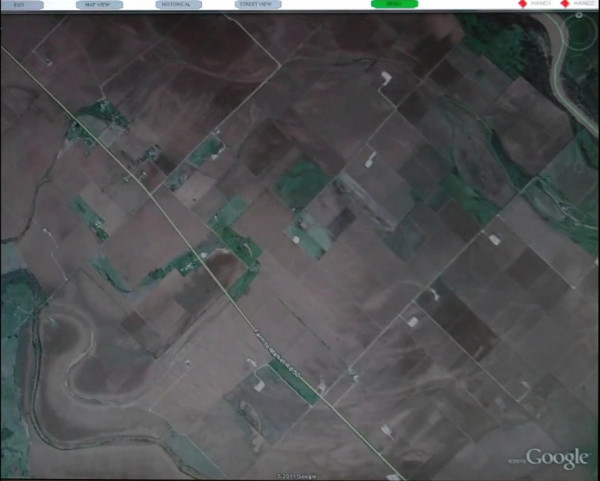
**Screenshot of Google Earth with Kinoogle's menu bar at the top of the screen**.

Kinoogle makes extensive use of third party software, which includes OpenNI drivers and NITE Middleware [9,10], OpenGL [45], and Google Earth [46]. OpenNI and NITE Middleware are both used for receiving and manipulating data obtained from the Kinect sensor, whereas OpenGL and Google Earth provide low-level graphics functionality and the front-end mapping application, respectively. OpenNI is a framework for utilising Natural User Interface (NUI) devices, and has abstractions which allow for the use of middleware to process images and depth information. This allows for user tracking, skeleton tracking, and hand tracking in Kinoogle. OpenNI is designed such that applications can be used independent of the specific middleware and therefore allows much of Kinoogle's code to interface directly with OpenNI while using the functionality from NITE Middleware. The main purpose of NITE Middleware is image processing, which allows for both hand-point tracking and skeleton tracking. NITE is responsible for sending the location (i.e., 3-D coordinates) of the hand points in every frame. Kinoogle also utilises OpenGL to create a graphical user interface (GUI) in the form of a menu bar at the top of the screen to provide visual feedback to the user (Figure 4). As a front-end, Kinoogle uses Google Earth, which provides visual imagery of Earth locations on a 3-D globe that the user is able to manipulate by panning, zooming, rotating, or tilting with an ordinary mouse, 3-D mouse (such as SpaceNavigator [47]) or keyboard.

### Kinoogle system software

**Figure 5:**
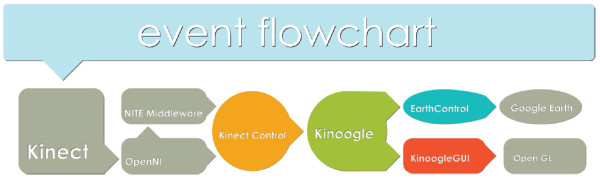
**Block diagram demonstrating the flow of data between the objects and third party software**.

At the top level, the system consists of four major objects, which serve as the connection to each of the third party software components; these four objects are: KinectControl, Kinoogle, EarthControl, and Kinoogle GUI (Figure 5). The system is based on hardware input, so it is designed to be event-driven (see 'code and documentation' folder in Additional files 1, 2).

### KinectControl

The first object, KinectControl, interfaces with NITE and OpenNI to collect relevant data from Kinect. It acts as a "plumber", placing certain objects into place to collect and forward point or skeleton data to Kinoogle. It also controls data flow from the Kinect hardware, such as halting incoming point/skeleton data.

The main loop in Kinoogle continuously calls KinectControl.kinectRun() function, which in turn makes three essential calls:

• context.WaitAndUpdateAll(), which forces OpenNI to get a new frame from Kinect and send this frame to NITE for processing;

• skeletonUpdater.run(), which looks at all the skeleton data contained in the UserGenerator node. If a skeleton is active, it sends all the joint position data to Kinoogle; and

• sessionManager.Update(), which forces sessionManager to process the hand point data contained in the current context and update all the point-based controls with callbacks.

The additional modules GestureListener and HandsUpdater register callbacks with PointControls and SessionManager. Thus, when Update() is called the callbacks are activated and the thread travels through these two modules on to Kinoogle and eventually to EarthControl.

Included in KinectControl is also one of the most important components, Fist Detector. It looks at the shape of the hand around the hand points, and determines whether the hand is closed or open. This information is then used by Kinoogle to engage and interact with Google Earth. FistDetector is based on calculating the area of the convex hull of the hand, and comparing it to the area of the hand. (The convex hull of a geometric shape is the smallest convex shape that contains all of the points of that shape.) In this case, Fist Detector allows us to quantify the area between open fingers, as if the hand were webbed. If the ratio of the hand to the hull is near one-to-one, the hand is most likely to be closed; if it is not one-to-one, the hand is most likely open.

### Kinoogle

Kinoogle's primary function is to interpret point data from Kinect Control in order to determine whether the user has produced specific gestures and poses for interacting with Earth Control and Kinoogle GUI. Kinoogle is responsible for the communication among the other three objects that control the third party software; this is achieved through using KinoogleMessage, which is contained in the Message object.

**Figure 6:**
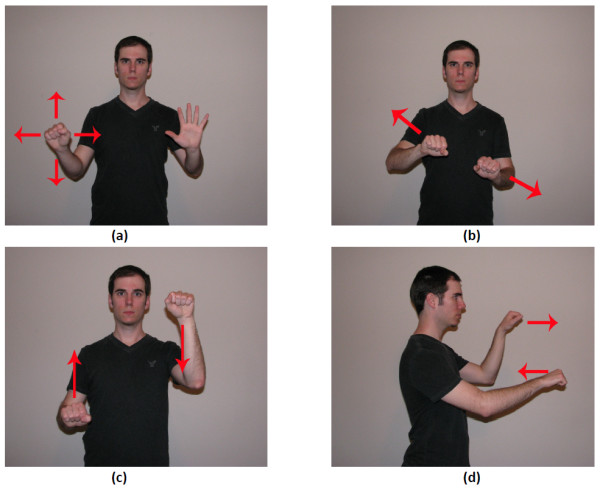
**Gesture movement used for (a) pan, (b) zoom, (c) rotate, and (d) tilt**.

Kinoogle also handles the detection of stationary poses, with which the user switches between various map modes (e.g., panning, zooming). Whenever the user activates his/her hands, an average location of the hands is calculated over the last 100 frames. An imaginary box (300 × 200 pixels) is then set around this average location. There are then three quadrants where the user can place his/her hands for pose detection: above the box, to the left, or to the right of the box. The various poses are activated when both hands are placed in corresponding quadrants around the average location. For example, placing one hand above the average location and the other hand to the right of the average location will be detected as the pose for tilt. Four map modes have been implemented (panning, zooming, rotation, and tilt), as follows:

• Panning is based on detecting the (x, y) position of either single hand when engaged (Figure 6(a)). A velocity variable is used to eliminate any slight movements that would result in flickering.

• Zooming requires a two-hand gesture (Figure 6(b)), and is performed by moving the hands either closer together or farther apart. Detection is based on calculating the distance between the hands to determine if they are moving together or apart.

• Rotation (in plane) also requires a two-hand gesture (Figure 6(c)), and detection is based on determining whether the hands move in opposite directions along the 'y' axis (i.e., vertical axis).

• Tilt (out-of-plane rotations) is also a two-hand gesture (Figure 6(d)) and is based on detecting whether the hands move in opposite directions along the 'z' axis (i.e., depth axis).

**Figure 7:**
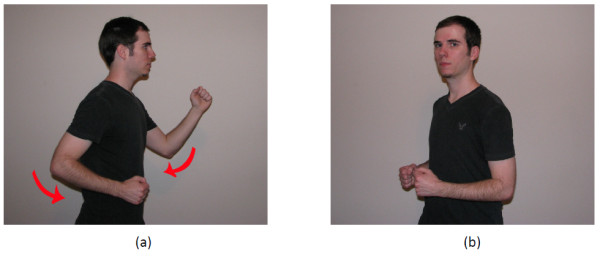
**Demonstration of (a) arms swinging and (b) shoulder twisting for Street View**.

Finally, Kinoogle is also responsible for interpreting skeleton data, which allows users to interact with Google Street View by means of intuitive "walking" and "turning" gestures. For walking detection, we focus on the speed by which the user swings his/her arms (Figure 7(a)); namely, we compute the average speed of the right and left arms based on the position of the elbows and compare it to a fixed threshold. Likewise, to control the camera angle in Street View, we detect whether the user is twisting his/her shoulders (Figure 7(b)); specifically, we determine which shoulder is closer to the Kinect sensor and then turn the camera view based on the difference between the two shoulders.

### EarthControl

EarthControl uses simulated mouse and keyboard actions to control Google Earth. The object receives EarthMessages from Kinoogle, e.g., earth. interpretMessage(&EarthMessage(Pan, panX, panY)), which it then interprets to determine the correct sequence of mouse or keyboard actions. By moving the pointer in certain axes and holding down certain mouse buttons, EarthControl effects the appropriate changes onto the map view. Other functions are executed by simulating the pressing of various keyboard buttons. The actual mouse and keyboard functions are performed through a series of functions that are grouped together in MouseLib. Specifically, MouseLib uses the Windows API (Application Programming Interface) call SendInput, which takes INPUT structures and inserts them into the operating system's (OS) input stream. These structures indicate either a mouse movement or key press. MouseLib is separate from EarthControl in order to ensure future OS portability of the Kinoogle system. As an example, the entire code that is specific to the Windows API is kept in MouseLib, so that an X-window or MacOSX version could be easily substituted in.

EarthControl also has two other noteworthy characteristics, which were created in response to specific issues with Google Earth: a mouse brake system, and a method for preventing a quick succession of single clicks. The mouse brake system prevents unwanted drift of the map view while panning. Initially, the left click button was released immediately when the hand becomes disengaged causing the map to continue to scroll, which made it almost impossible to leave the map in a stationary position. The solution to this problem was to measure the speed of the mouse pointer when the hand is disengaged. If the mouse pointer moves under a certain speed, the "brake" engages, leaving the mouse button clicked down and the pointer stationary for a certain amount of time. The second method prevents accidental double-clicks of the left mouse button by starting a timer after the first left mouse click and not allowing another to happen until after the timer is completed.

### Kinoogle GUI

**Figure 8:**
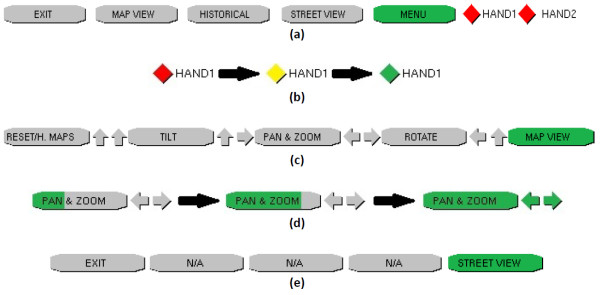
**Kinoogle GUI bar**. (a) The Kinoogle status window, as seen immediately at program start, before hand tracking is initialised. (b) The colour changes between red, yellow, and green based on hand detection. (c) The Kinoogle status window, as seen in Map Mode without any sub-mode selected. (d) Progress bar charging while a pose is detected. (e) The Kinoogle status window, as it appears while in Street View.

Kinoogle GUI provides the user interface to the system, and is implemented as a menu bar on top of the application (Figure 4). Kinoogle GUI runs as a separate thread from the main Kinoogle thread, from which it receives commands through GUIMessages, which is part of the Message object. The menu interface (window and buttons) is implemented with an OpenGL render object. There are three sections of information drawn on the menu bar for the user (Figure 8(a)). The first section shows the different features that are available in the current mode, as well as arrows corresponding to the pose to activate a given feature. The second section is a single button used to show the user the current mode. The third section contains two diamonds representing user's hands. Additional details about the menu interface are provided below under 'User manual: Operating instructions'.

### User manual

#### Hardware and software installation

Kinoogle requires a Microsoft Kinect, which is currently available worldwide for purchase from multiple retailers. The Kinect package contains the Kinect sensor, in addition to a power adapter with an electrical plug and a USB connector (Figure 1). Kinect requires more power than a USB port can provide, so the electrical plug must be inserted into an electrical outlet before using the device. The USB connector must be inserted into the computer where Kinoogle will run (Kinect should be plugged in to the PC's USB port only *after *OpenNI/NITE driver installation is complete--see below). Kinoogle also requires third party drivers and software to be installed on the host computer. The download links for the Windows OS version of these software packages are: Google Earth [48], OpenNI [49], and NITE [50] (the x86 (32-bit) versions of OpenNI and NITE work well under both 32-bit and 64-bit (x64) versions of Microsoft Windows). Additional files 1, 2 are a compressed archive containing Kinoogle's installation package for Windows. It has been tested on several machines to make sure it works robustly.

#### Operating instructions

The user must ensure that Google Earth is running before starting Kinoogle. Once Kinoogle is started, the status indicator shown in Figure 8(a) will become visible at the top of the screen. Two coloured diamonds on the right-hand-side indicate the status of the program's hand tracking: (i) red indicates that the corresponding hand has not been detected, (ii) yellow indicates that the hand has been detected but is not engaged, and (iii) green indicates that the hand is detected and also engaged; see Figure 8(b). Hands are engaged when clenched into a fist.

To initialise hand tracking, the user waves one hand until the status indicator for HAND1 becomes yellow, followed by waving the other hand near the location of the first hand until the indicator for HAND2 also becomes yellow. The user may use either hand to initialise tracking. Once hand tracking has been initialised, the program will automatically enter Map Mode, and the menu bar will change to reflect this; see Figure 8(c).

**Figure 9:**
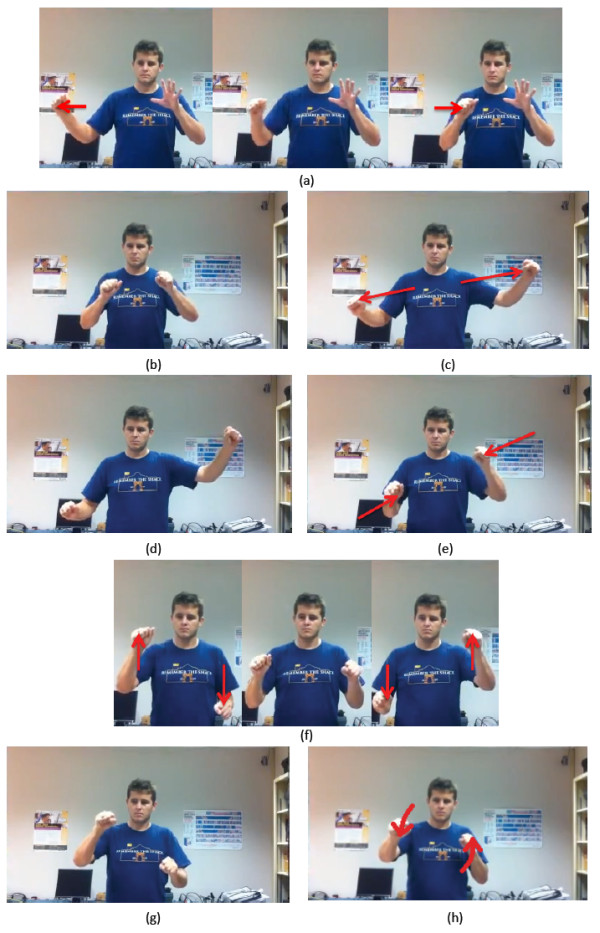
**Procedures for panning, zooming in and out, rotating and tilting the map**. (a) Procedure for panning. The map can be panned both directions vertically. (b, c) Procedure for zooming in: (b) the user moves hands together and engages them, then (c) moves them apart. (d, e) Procedure for zooming out: (d) the user moves hands apart and engages them, then (e) brings them together. (f) Procedure for rotating the map. (g, h) Procedure for tilting the map: (g) the user engages the hands and then (h) moves one forward, and one to the back. See Figure 6(d) for alternative view.

**Figure 10:**
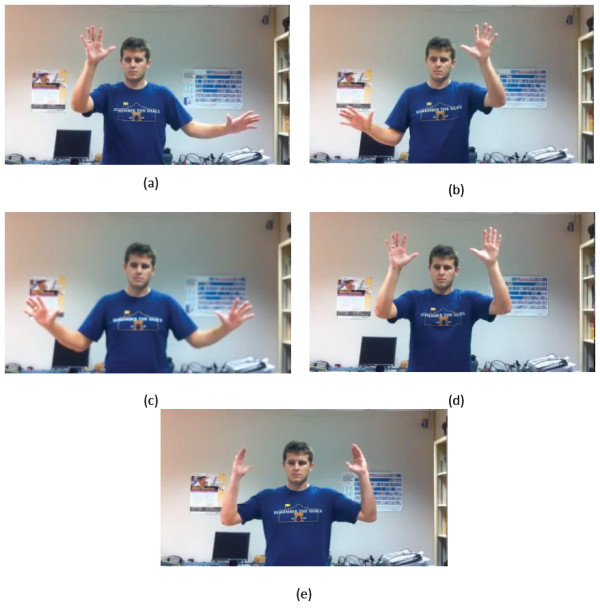
**Poses required to switch among sub-modes**. (a) To enter Rotate Mode, the user holds his/her right hand up and moves his/her left hand to his/her left. (b) To enter Tilt Mode, the user holds his/her left hand up and moves his/her right hand to his/her right. (c) To enter Pan/Zoom Mode, the user holds both hands to the side. (d) To reset the map, the user holds both hands up at the same level as his/her head. (e) The touchdown pose is used to calibrate Kinoogle for Street View.

Map Mode has four sub-modes, each one representing a different form of map manipulation:

1. Pan/Zoom: This is the default sub-mode when first entering Map Mode. This sub-mode allows the user to scroll the map in any direction, as well as zoom in and out. To scroll the map, the user engages one hand, and moves that hand as if he/she were dragging the map on a surface; see Figure 9(a). To zoom in, the user un-engages both hands and brings them together, then engages both hands and pulls them apart; see Figure 9(b, c). Zooming out is performed by un-engaging both hands and moving them apart, then engaging both hands and bringing them together; see Figure 9(d, e).

2. Rotate: This sub-mode allows the user to adjust the rotation angle of the map on the x, y plane. To rotate the map, the user engages both hands, and moves them in opposite vertical directions, i.e., using a motion similar to turning a steering wheel; see Figure 9(f).

3. Tilt: This sub-mode allows the user to rotate the map out-of-plane. To tilt the map, the user engages both hands and moves them in opposite z directions, i.e., using a motion similar to rotating a crankset (chainset); see Figure 9(g-h).

4. Reset Map/Historical Maps: The map can be reset by un-engaging both hands and bringing them up to the same level as user's head; see Figure 10(d). This will also set up the Historical Maps sub-mode by moving the cursor to the time period selector. To change the time period, the user engages one hand and moves the slider to the desired time period. Note that the available time periods will change depending on the area being viewed. When entering the Historical Maps sub-mode, the map's tilt and rotation angles will be reset.

To switch between sub-modes, the user must make and hold a specific pose for a short time (Figure 10). The arrows to the right of each box in Figure 8(c) indicate how the user must hold his/her hands in order to enter that mode. A progress bar for the target mode will fill as the user holds the pose; see Figure 8(d).

**Figure 11:**
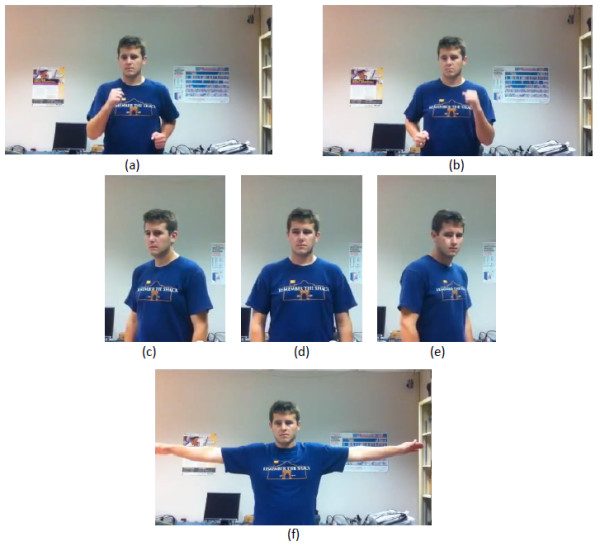
**Poses in Street View**. (a, b) Walking forward. (c-e) Turning right, facing straight and turning left, respectively. (f) Exiting Street View.

Kinoogle also allows the user to interact with Google Earth in the Street View mode. This mode is entered by using the Pan/Zoom mode to zoom in as much as possible into a street that is enabled for Street View. Google Earth will automatically zoom down to the street. Once the map is zoomed onto the street, the user should make a touchdown pose to calibrate Kinoogle for Street View; see Figure 10(e). The status indicator labels will also change, as illustrated in Figure 8(e). While in Street View, all the controls for Map Mode are disabled. Street View mode has three main sub-modes that allow you to interact with the map:

• Walking: To move forward, the user swings his/her arms while standing in place. The user does not have to move his/her legs, although he/she may walk in place if desired; see Figure 11(a, b).

• Turning: The user is able to change camera views by twisting his/her shoulders towards or away from the camera. The user must ensure that he/she is only twisting his/her shoulders, and not turning his/her entire body; see Figure 11(c-e).

• Exit: To exit the Street View mode, the user extends both arms straight out horizontally; see Figure 11(f). After exiting Street View, the map will zoom out. The user must then reinitialise the hand detection in order to re-enter Map Mode and continue manipulating the map.

## Discussion and conclusions

Nowadays, online 3-D virtual globes are commonly used to visualise and explore spatial epidemiology and public health data [51-55]. Users normally employ one or more conventional input devices (mouse, 3-D mouse and/or keyboard) to navigate online virtual globes. Hands-free gesture and speech recognition, e.g., as offered by the Kinect sensor, are expected to change our human-computer interaction with online and desktop interfaces over the coming years [56,57]. Improvements in the way we interface with virtual globes are always welcome, provided that compelling 'use scenarios' can be conceived to justify recommending and investing in them over or alongside existing PC input methods.

Kinoogle offers a good example of a natural interface with Google Earth and Street View using only body movements and gestures, without having to touch any physical input device (the user's body becomes the controller). Kinoogle can be greatly improved if it can also make use of the advanced speech recognition functionality afforded by the Kinect device as an alternative method for navigating Google Earth using voice commands besides hand and body gestures (e.g., where a person lacks the necessary physical agility and dexterity to perform certain "awkward" gestures or where more fine or quicker control of the interface is required than what can be achieved with body gestures alone).

Other possible Kinoogle improvements include adding a Kinect-controlled virtual on-screen keyboard for text input (e.g., to type in the 'Fly To' box of Google Earth), similar to the one provided by KinClick [58] for the KinEmote package that runs under Windows [59], and perhaps developing special versions of 3-D virtual globes with larger interface elements, big icons, and Kinect-friendly dialog boxes or tabs to make it easier for Kinect users to navigate and select/de-select on-screen items and options (e.g., virtual globe 'Layers' in Google Earth).

The hands-free convenience of gesture and speech recognition can prove extremely useful in a number of practical and exclusive 'use scenarios' where mouse or multi-touch screen inputs are difficult, such as when delivering presentations involving 3-D virtual globes on a large screen on stage to a large audience, e.g., this presentation of Microsoft's WorldWide Telescope using the Kinect sensor: [60]. (Kinect can also support two simultaneous active users/presenters.)

Collaborative GIS in virtual situation rooms involving distributed teams of users [61] is another 'use scenario' that can benefit from Kinect's unique features such as its headset-free 3-D and enhanced video teleconferencing functionalities [23-26], in addition to its use as 3-D motion sensing/gestures and speech recognition NUI for controlling a shared 3-D virtual globe during networked spatial data presentations [39,40]. Using the Kinect sensor to create 3-D maps of real world locations and objects [62,63] is still not very refined for serious use, but might soon get added to the GIS professional's toolkit as the technology evolves and matures.

Kinect can be an excellent, very usable and entertaining way for navigating Google Street View and exploring new places and cities (virtual tourism). The visual feedback in the form of the changing street scenery as the user walks (virtually, as on a treadmill [64]) in Street View can be used to encourage people to do physical exercise and burn calories with the Kinect device for prolonged periods of time, without getting quickly bored (e.g., as part of obesity management and prevention programmes). This use of Kinect for physical fitness purposes is already implemented as a game for the Xbox 360 game console [65].

Hands-free gesture and speech recognition NUIs are not a full replacement for more conventional interaction methods; for example, not all users have the necessary muscular agility and dexterity, physical room space, or large enough screen size (for comfortable viewing from the user's minimum distance away from the sensor) to use a Kinect sensor, and more conventional navigation of virtual globes with a 3-D mouse [47] or a multi-touch screen can sometimes offer more precise and smooth control of the 3-D map. However, depth sensors such as Microsoft Kinect, ASUS Xtion PRO/PRO Live [66], and Lenovo iSec [67] remain an interesting alternative or complementary method of interaction with 3-D virtual globes, with some exclusive applications where these devices cannot be easily matched (e.g., hands-free virtual globe presentations to large audiences) and other applications where the NUI affordances of these sensors can greatly improve a user's experience (e.g., when interacting with large and stereoscopic screens at a distance [36,68]).

## Competing interests

The authors declare that they have no competing interests.

## Authors' contributions

MNKB conceived and drafted the manuscript with contributions (description of Kinoogle) from BJB, CW, JM, AT, and RG-O. MNKB also conducted the literature review for this article, proposed the 'use scenarios' for a Kinect-based NUI for 3-D virtual globe navigation, and wrote all of the 'Background' and 'Discussion and conclusions' sections. BJB, CW, and JM conceived and coded Kinoogle as their CSCE 483 course project at Texas A&M University, with input from AT (CSCE 483 course Teaching Assistant) and under the supervision of RG-O (CSCE 483 course Instructor). All authors read and approved the final manuscript. Commercial products and company/brand names mentioned in this paper are trademarks and/or registered trademarks of their respective owners.

## Supplementary Material

Additional file 1 **Installation package for Kinoogle (part 1 of 3)**. Compressed (zipped) archive containing Kinoogle's installation package for Microsoft Windows operating systems. Download and unzip the contents of Additional file 1, Additional file 2, and Additional file 3 to the same hard drive location, then run 'Additional_file.part1.exe' from that location. Click here for file

Additional file 2 **Installation package for Kinoogle (part 2 of 3)**. Compressed (zipped) archive containing Kinoogle's installation package for Microsoft Windows operating systems. Download and unzip the contents of Additional file 1, Additional file 2, and Additional file 3 to the same hard drive location, then run 'Additional_file.part1.exe' from that location. Click here for file

Additional file 3 **Installation package for Kinoogle (part 3 of 3)**. Compressed (zipped) archive containing Kinoogle's installation package for Microsoft Windows operating systems. Download and unzip the contents of Additional file 1, Additional file 2, and Additional file 3 to the same hard drive location, then run 'Additional_file.part1.exe' from that location. Click here for file
